# Microvesicles Derived from Human Umbilical Cord Mesenchymal Stem Cells Enhance Alveolar Type II Cell Proliferation and Attenuate Lung Inflammation in a Rat Model of Bronchopulmonary Dysplasia

**DOI:** 10.1155/2022/8465294

**Published:** 2022-06-27

**Authors:** Ou Zhou, Jingyi You, Xiaochuan Xu, Jiang Liu, Huijun Qiu, Chang Hao, Wenjing Zou, Wenjie Wu, Zhou Fu, Daiyin Tian, Lin Zou

**Affiliations:** ^1^Department of Respiratory Medicine, Children's Hospital of Chongqing Medical University, National Clinical Research Center for Child Health and Disorders, Ministry of Education Key Laboratory of Child Development and Disorders, Chongqing Key Laboratory of Pediatrics, Chongqing Engineering Research Center of Stem Cell Therapy, Chongqing 400014, China; ^2^Department of Pediatrics, Chongqing Youyoubaobei Women and Children's Hospital, Chongqing 401122, China; ^3^Center of Clinical Molecular Medicine, Children's Hospital of Chongqing Medical University, Chongqing 400014, China; ^4^Clinical Research Unit, Children's Hospital of Shanghai Jiaotong University, Shanghai 200062, China

## Abstract

Although it is known that exosomes derived from human umbilical cord mesenchymal stem cells (hUCMSCs) alleviate hyperoxic lung injury of bronchopulmonary dysplasia (BPD) in animal models, the role of microvesicles (MVs) derived from hUCMSCs in BPD is poorly defined. Furthermore, antenatal inflammation has been linked to high risk of BPD in preterm infants. The purpose of this study was to explore whether MVs derived from hUCMSCs can preserve lung structure and function in an antenatal lipopolysaccharide- (LPS-) induced BPD rat model and to clarify the underlying mechanism. We demonstrate that antenatal LPS induced alveolar simplification, altered lung function, and dysregulated pulmonary vasculature, which restored by hUCMSCs and MVs treatment. Furthermore, MVs were large vesicles with a diameter of 100-900 nanometers and mostly uptaken by alveolar epithelial type II cells (AT2) and macrophages. Compared with the LPS-exposed group, MVs restored the AT2 cell number and SP-C expression *in vivo* and promoted the proliferation of AT2 cells *in vitro*. MVs also restored the level of IL-6 and IL-10 in lung homogenate. Additionally, PTEN/AKT and MAPK pathways were associated with the protection of MVs. Taken together, this study suggests MVs derived from hUCMSCs improve lung architecture and function in an antenatal LPS-induced BPD rat model by promoting AT2 cell proliferation and attenuating lung inflammation; thus, MVs provide a promising therapeutic vehicle for BPD treatment.

## 1. Introduction

Bronchopulmonary dysplasia (BPD) is a serious and common chronic lung disease of premature infants. It is caused by the imbalance between lung injury and repair in the developing immature lung [1]. BPD is characterized by stunted alveolar development, dysregulated pulmonary vasculature, altered lung function, and pulmonary hypertension (PH) [2]. Despite improvements in perinatal care, the incidence of BPD has not been reduced. BPD remains the most common late morbidity and the most frequent complication of extremely preterm birth [3, 4].

The cause of BPD is associated with a wide variety of risk factors [5]. Historically, hyperoxia, invasive mechanical ventilation, and sepsis have been considered the key contributors to BPD [6]. However, with the increasing survival rate of the most extremely preterm infants [7], prematurity and low birth weight have become the strongest risk factors of BPD and they are strongly related to antenatal determinants [8–15]. Therefore, there has been a growing recognition that the early identification and treatment of preterm infants at high risk of BPD may provide greater opportunities to prevent and alleviate BPD [16]. However, in preclinical studies, the most commonly used animal model for BPD research involves prolonged exposure to postnatal hyperoxia. Several studies have reported that antenatal injection of LPS causes impaired alveolar development and dysregulated vasculature by inducing inflammation to mimic features of human BPD, even in the absence of postnatal hyperoxia or mechanical ventilation; thus, antenatal LPS exposure may be a better model to reflect the influence of antenatal factors on BPD [16–18].

Human umbilical cord mesenchymal stem cells (hUCMSCs), with low immunogenicity and easy accessibility, are effective for inflammatory disease treatment and regenerative medicine, which bring new hope to patients with BPD [19]. In preclinical studies, our group has previously revealed the beneficial effect of hUCMSCs on alleviating BPD in an animal model of exposure to postnatal hyperoxia [20, 21]. In clinical studies, many phase 1 clinical trials have reported preliminary data regarding the safety and potential efficacy of MSC treatment for lung injury [22, 23]. MSC transplantation in preterm infants at high risk of BPD is safe, with no adverse respiratory, growth, and neurodevelopmental effects [24]. Furthermore, MSCs have been shown to exert their beneficial effects *via* paracrine mechanisms, and MSC-derived conditioned medium revealed a comparable therapeutic effect [25]. As a major paracrine mediator of MSCs, extracellular vesicles (EVs)—especially exosomes (also called small EVs)—have been reported to attenuate hyperoxia-induced lung injury through treatment mechanisms that include three aspects: anti-inflammatory processes [26], proangiogenesis [27, 28], and modulation of lung macrophage phenotype [29]. Besides exosomes, microvesicles (MVs, also called large EVs)—another type of EVs that originate from cell membranes, with sizes ranging from 150 to 1000 nm [30]—have been reported to mediate the modulation of immune interactions, anti-inflammatory processes, and angiogenesis, considering that they contain proteins, RNA, miRNA, and trophic factors derived from parent cells [31, 32]. One study reported that human MSC-derived MVs could alleviate lung injury induced by bacterial pneumonia [33]. Another study found that MVs promoted angiogenesis on human umbilical vein endothelial cells *in vitro* [34]. However, the therapeutic effects of MVs on BPD remain largely unknown.

In this study, we aim to explore whether MVs derived from hUCMSCs can preserve lung development and the underlying mechanism in an antenatal LPS-induced BPD rat model. We found that MVs derived from hUCMSCs were able to enhance alveolar development by promoting AT2 cell proliferation and ameliorating inflammation. Our findings provide insights into the paracrine effects of MSCs on the antenatal rat model of BPD.

## 2. Materials and Methods

### 2.1. Animals

Sprague-Dawley (SD) rats were purchased from the Experimental Animal Center of Chongqing Medical University and maintained at the Animal Laboratory Center of Pediatrics, Children's Hospital of Chongqing Medical University. All animal procedures and protocols were approved by the Ethics Committee of Children's Hospital of Chongqing Medical University. The animals were housed under controlled temperature (22 ± 1°C) with a 12-hour day/night cycle with food and water ad libitum. Neonatal mortality was checked daily.

### 2.2. Intra-Amniotic (IA) Injections of LPS and Antenatal Rat Model of BPD

**Figure 1:**
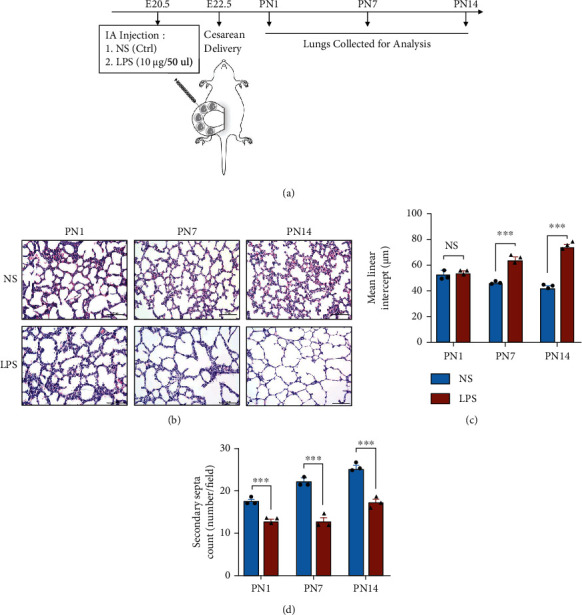
Establishment and characterization of bronchopulmonary dysplasia (BPD) model induced by intra-amniotic injection of LPS (IA-LPS). (a) The schedule involved experimental BPD rat model caused by intra-amniotic (IA) injection of normal saline (NS) or LPS and time points to collect lungs. (b) Representative lung sections stained with H&E on postnatal day 1 (PN1), PN7, and PN14, scale bar = 100 *μ*m. Quantification of the (c) mean linear intercept (MLI) and (d) secondary septa (*N* = 3, *t*-test, NS: not significant, ^∗∗∗^*P* < 0.001).

As shown in Figure 1(a), pregnant female Sprague-Dawley rats (8- to 10-week old) received IA injections of LPS on day 20.5 of gestation (term = day 22.5 of gestion) in accordance with a previous report [35]. Briefly, laparotomy was performed on each dam under general anesthesia with isoflurane inhalation. After anesthesia, amniotic sacs were fully exposed from the abdominal cavity; IA injections were started from the amniotic sac closest to the right ovary and were administered to up to 10 sacs per dam in a counter-clockwise sequence. Pregnant rats were randomly assigned to receive normal saline (NS; 50 *μ*L per amniotic sac) for the control group or LPS (10 *μ*g of *Escherichia coli* 055: B55 diluted with 50 *μ*L NS per sac) for the antenatal BPD model. Two days following IA injections, cesarean section was performed on pregnant rats under general anesthesia. All the rat pups (male and female) in the injected amniotic sacs were delivered and then placed with foster mother rats.

### 2.3. Preparation and Identification

Exosome-free fetal bovine serum (FBS) was prepared overnight using ultracentrifugation at 4°C, 120000 × g for 12 hours. MVs were harvested from the medium of hUCMSCs, obtained from the Chongqing Stem Cell Engineer Research Center. Briefly, P4-P6 HUCMSCs were seeded at 1 × 10^5^ cells per 100 mm dish and cultured for 48 hours in 10 mL DF-12 with 10% exosome-free FBS. The medium was collected and centrifuged at 4°C, 400 × g for 5 minutes and 1500 × g for 10 minutes to remove cells and debris. The supernatant was ultracentrifuged at 18000 × g for 30 minutes to pellet the MVs. The total protein concentration of the MVs was measured using BCA kit (Beyotime, China) as per the manufacturer's recommendations. The isolated MVs were stored at −80°C for later use and characterized by TEM (Hitachi, S-3000N, Japan) and Zetasizer Nano S90 (Malvern, UK).

### 2.4. hUCMSCs or MV Administration and Tissue Collection

**Figure 2:**
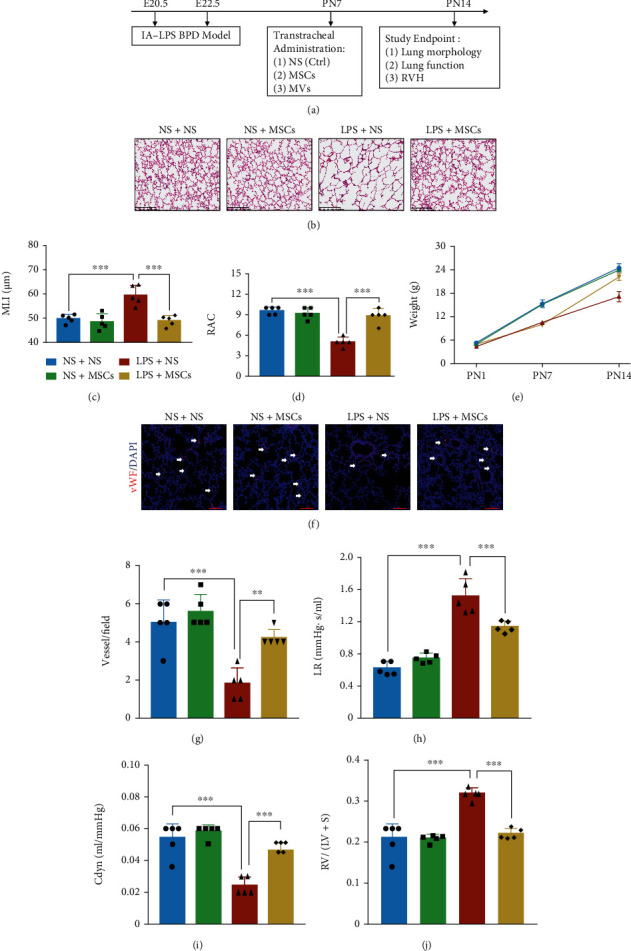
hUCMSC treatment attenuates lung morphology and function in the IA-LPS BPD model. (a) The study design represents the IA-LPS BPD model and hUCMSC treatment. Transtracheal administration of hUCMSCs was conducted on PN7. Study end points on PN14 involved analysis of lung morphology, function, and right ventricular hypertrophy (RVH). (b) Representative lung sections stained with H&E on PN14, scale bar = 200 *μ*m. (c) Quantification of MLI (*N* = 5, ANOVA, ^∗∗∗^*P* < 0.001). (d) Quantification of radial alveolar counts (RAC) (*N* = 5, ANOVA, ^∗∗∗^*P* < 0.001). (e) Comparison of pups' weight among four groups (*N* = 5, ANOVA). (f) Representative immunofluorescence images of vWF staining in the lung on PN14 in each group, scale bar = 100 *μ*m. (g) Quantification of vWF-positive vessels (<100 *μ*m) (*N* = 5, ANOVA, ^∗∗^*P* < 0.01 and ^∗∗∗^*P* < 0.001). (h) Lung resistance (LR) and (i) dynamic compliance (Cdyn) were determined from anesthetized and ventilated pups (*N* = 5, ANOVA, ^∗∗∗^*P* < 0.001). (j) The right ventricular index was determined by RV/LV + *S* to measure RVH (*N* = 5, ANOVA, ^∗∗∗^*P* < 0.001).

On postnatal day 7 (PN7), as illustrated in Figure 2(a), the treatment groups received 40 *μ*L hUCMSCs (1 × 10^6^ cells per pup) or MVs by intratracheal route, while the control groups received 40 *μ*L of intratracheally administered NS. On PN14, the pups were anesthetized with an intraperitoneal injection of pentobarbital sodium (40 mg/kg). Then, the thoracic cavity was opened, and the lungs were perfused with cold PBS through the heart. The lungs were harvested for histological assessment. The hearts were weighed to determine RVI. Body weight was measured at birth, on PN7, and on PN14. Animals were randomized into different groups using table of randomized numbers. Treatments were administered in a blinded manner. In order to minimize the cage effect, pups among groups were divided and housed in 4-4 cages, with five to eight pups per cage.

### 2.5. Lung Alveolarization Assessment

One 4 mm thick transverse section was taken from the midplane of the left lobe of the fixed lungs per animal and processed and embedded in paraffin wax. All the sections were stained with hematoxylin and eosin, and alveolarization was assessed by performing radial alveolar counts (RAC), secondary septa count, and median linear intercepts (MLIs) as previously described [21].

### 2.6. Pulmonary Vasculature Assessment

Pulmonary vasculature was measured by immunofluorescence with von Willebrand factor (vWF). Fixed left lungs were embedded in glue (Sakura, Japan) and cut with a microtome at 10 *μ*m at −20°C (Leica CM1950, Germany). Frozen sections and cells were then fixed with 4% PFA and blocked with 10% BSA. Subsequently, the samples were stained with anti-vWF antibody (1 : 100, PA5-80223, Thermo Fisher Scientific, USA), washed, and incubated with goat anti-mouse Fluor cy3-conjugated secondary antibody (Proteintech, USA). Cell nuclei were counterstained with DAPI for 15 minutes, and fluorescence was observed on a Leica laser confocal microscope (C2+ system, Nikon, Japan). Five random images were captured at 200x magnification for quantification of vWF.

### 2.7. Lung Function and Right Ventricular Hypertrophy

Lung function was determined in PN14 pups with a computer-controlled small-animal ventilator (Emka, USA). Briefly, the rats were anesthetized with pentobarbital sodium (40 mg/kg), intubated following tracheostomy, and mechanically ventilated at a rate of 150 breaths/min, with a tidal volume of 8 mL/kg and a positive end-expiratory pressure (PEEP) of 3 cm H_2_O with the computer-controlled small-animal ventilator (Emka, USA). LR and Cdyn were recorded every five seconds. Right ventricular hypertrophy was determined by the right ventricular index (RVI), which represents the weight of right ventricle relative to left ventricle+septum (RV/(LV + *S*)). Briefly, after removing the arterial and adipose tissue on the epicardium, we collected and weighed the left ventricle plus the interventricular septum and the right ventricle tissue by cutting along the edge of the ventricle and the interventricular septum.

### 2.8. MV Localization in the Lungs of Antenatal LPS-Induced BPD Rats

MVs were labeled with a DiO Green Fluorescent (Beyotime, China) as per the manufacturer's protocol. Immunofluorescence localization of donor MVs was performed on 10 *μ*m thick cryostat sections on PN9 (48 hours after MV administration). The following primary antibodies were used as markers of alveolar epithelial type I cells (AT1), AT2, vascular endothelial cells, vascular pericytes, total macrophages, and smooth muscle cells: Aquaporin-1 (AQP1, 1 : 200, Abcam), prosurfactant protein C (SP-C, 1 : 100, Abcam), CD31 (1 : 100, Abcam), NG2 (1 : 200, Abcam), Iba-1 (1 : 200, Abcam), and *α*-smooth muscle actin (*α*-SMA) (1 : 200, Abcam), respectively. Then, Cy-3 dye-labeled IgG was used as the secondary antibody (Beyotime, China). Fluorescence was observed on Leica laser confocal microscopy (C2+ system, Nikon, Japan), and at least five different visual fields were randomly selected from each sample.

### 2.9. Western Blotting

The lung tissues were harvested in a lysis buffer (25 mMTris-HCl (pH 7.5), 137 mM NaCl, 2.7 mM KCl, and 1% Triton X-100) containing protease and phosphatase inhibitor cocktail (Sigma-Aldrich, St. Louis, MO). The protein concentration was measured using BCA protein assay reagent (Beyotime, China). Equal amounts of proteins were separated using SDS-PAGE and transferred to polyvinylidene difluoride membranes (Thermo Scientific, Rockford, IL). The membranes were blocked with 5% skim milk in PBS containing 0.1% Tween 20 (PBS-T) for one hour and then incubated with the specified antibodies. Signals were detected using the ECL detection system (Gene Company Limited, Hong Kong, China).

### 2.10. Statistical Analysis

Statistical analysis was performed with the GraphPad Prism software (Version 5.0, San Diego, CA, USA). A *t*-test was used for statistical comparisons between two groups, and a one-way analysis of variance (ANOVA) with Kruskal–Wallis/Dunns post hoc test was applied for significance testing among more than two groups. Investigators were blinded to experimental groups for histological analysis and physiological measurements. Statistical significance is indicated as follows: ^∗^*P* < 0.05, ^∗∗^*P* < 0.01, and ^∗∗∗^*P* < 0.001. NS means no significance. Data are presented as mean ± standard error.

## 3. Results

### 3.1. Establishment and Characterization of an Antenatal LPS-Induced BPD Model

Considering that antenatal LPS injection could cause impaired alveolar structure and dysregulated vasculature to mimic human BPD features [35], we established the rat model of BPD by IA injection of LPS and cesarean delivery. IA LPS lead to 88 ± 3% survival of rat pups on PN1, and no more pups dead on PN7 and PN14. The pups' lungs were collected for analysis on PN1, PN7, and PN14 (Figure 1(a)). Compared with the NS control (NS Ctrl), the lung structures in the LPS group had typical characteristics of alveolar simplification (Figure 1(b)). The MLI in antenatal LPS without hyperoxia on PN7 and PN14 was significantly higher than that in the NS group, although without statistical difference on PN1 (Figure 1(c)). The secondary septa were significantly reduced in the LPS group compared with the NS group on PN1, PN7, and PN14 (Figure 1(d)), demonstrating the successful establishment of the rat model of BPD induced by intra-amniotic injections of LPS (IA-LPS BPD model).

### 3.2. hUCMSC Treatment Improves Lung Development and Alleviates RVH in IA-LPS BPD Model Rats

Given that there is currently no report on the effects of hUCMSCs on IA-LPS BPD model, we first examined the effect of hUCMSCs on the antenatal rat model of BPD induced by antenatal LPS. The pups received hUCMSCs on PN7 by transtracheal administration (Figure 2(a)). We found that compared with the rats exposed to LPS alone, the lung development was improved on PN14, the MLI was significantly decreased, and the RAC was increased by hUCMSC treatment (Figures 2(b)–2(d)). Although all the groups had the same birth weight, rats exposed to LPS had a slow weight gain, but there was a catch-up growth period after transtracheal administration of hUCMSCs (Figure 2(e)). Small vessels (<100 *μ*m) in rats exposed to LPS were significantly less abundant than those in the NS group; however, the abundance of small vessels increased after hUCMSC treatment, compared with the LPS group (Figures 2(f) and 2(g)). The pulmonary function results showed that hUCMSC treatment reduced lung resistance, increased lung compliance, and decreased RVH as compared with rats exposed to LPS alone (Figures 2(h)–2(j)).

### 3.3. Characterization of hUCMSC-Derived MVs

**Figure 3:**
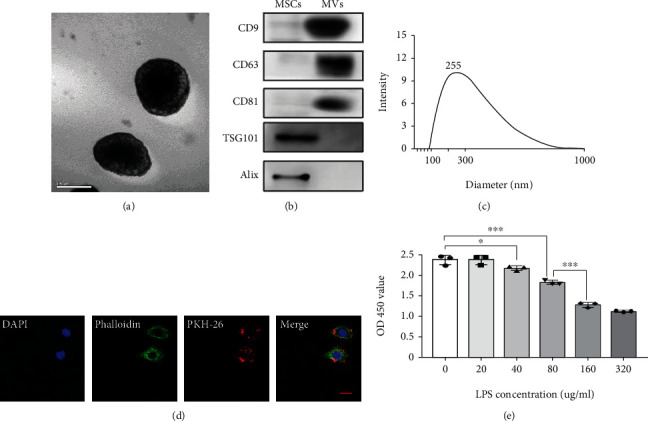
Characterization of hUCMSC-derived MVs. (a) Representative images of transmission electron microscopy (TEM) for MVs derived from hUCMSCs, scale bar = 500 nm. (b) Detection of MV-specific biomarkers for both analyzed MVs and whole-cell lysates by western blot. (c) Nanoparticle tracking analysis (NTA) measurement of the mean size of MVs. (d) Representative images showing the colocalization of phalloidin immunostaining (green) with internalized MVs (red), scale bar = 50 *μ*m. (e) MLE-12 viability after exposure to different concentrations of LPS in vitro (*N* = 3, ANOVA, ^∗^*P* < 0.05 and ^∗∗∗^*P* < 0.001).

Considering that MVs derived from hUCMSCs have multiple regenerative roles in skin repair and other diseases [31, 32], we isolated MVs from hUCMSCs by ultracentrifugation and characterized them by electron microscope observation and nanosizer measurement. According to scanning electron microscopy, the MVs showed a double membrane structure (Figure 3(a)), and WB confirmed the expression of MV markers, CD9, CD63, CD81, TSG101, and Alix (Figure 3(b)). Nanosight analysis showed that the diameter of the MVs ranged from 100 to 900 nm, with the main peak at 255 nm (Figure 3(c)). The total protein content harvested from 200 mL supernatant was 165.2 ± 5.7 *μ*g (data not shown). Subsequently, we determined whether MVs can be internalized into the MLE-12 cells (an AT2 cell line). MVs labeled with red fluorescence PKH26 colocalized with the green fluorescence signals of cytoskeleton stained with phalloidin, indicating the internalization of MVs into the AT2 cells (Figure 3(d)). We then performed mimic infection injury *in vitro* using LPS in the following experiments, so the effects of a series of differing LPS concentrations on MLE-12 cells were evaluated. Treatment with LPS at a concentration higher than 160 *μ*g/mL for 48 hours obviously inhibited AT2 proliferation (Figure 3(e)). Therefore, 80 *μ*g/mL of LPS was selected for the subsequent *in vitro* experiments.

### 3.4. MVs Improve Lung Structure and Prevent Loss of Lung Function in IA-LPS BPD Model

**Figure 4:**
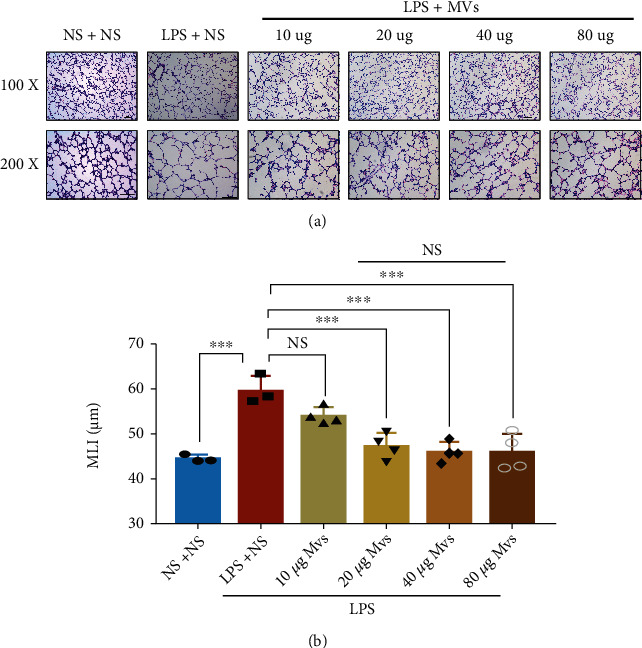
MVs improve antenatal LPS-induced lung injury in a dose-dependent manner. (a) Representative lung sections stained with H&E on PN14, scale bar = 100 *μ*m. (b) Quantification of the MLI (*N* = 3‐4, ANOVA, NS: not significant, ^∗∗∗^*P* < 0.001).

**Figure 5:**
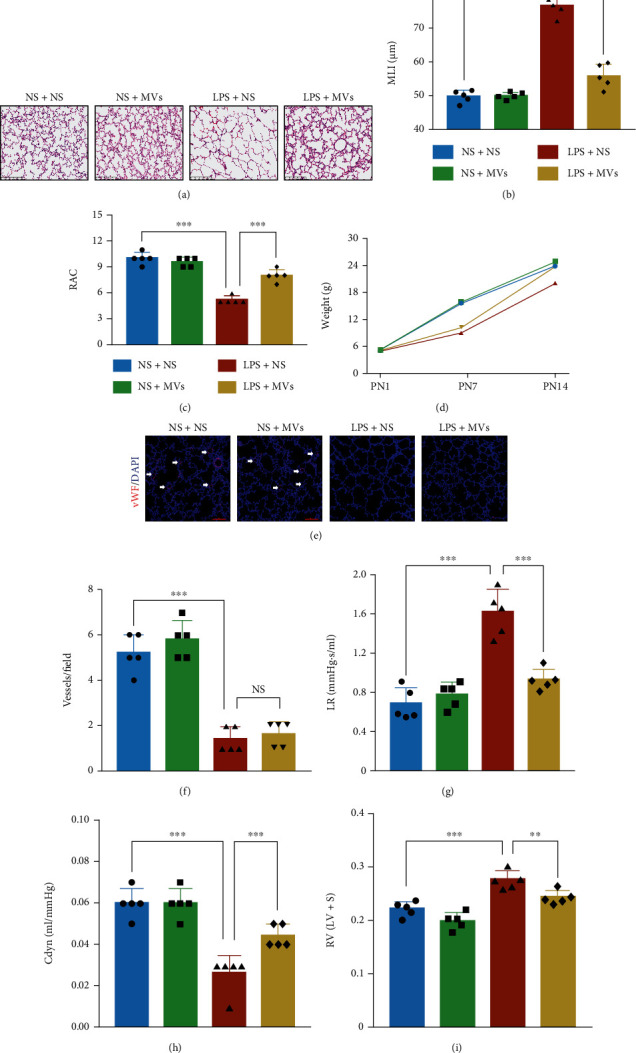
MVs improve lung development and function in the IA-LPS BPD model. (a) Representative lung sections stained with H&E on PN14, scale bar = 200 *μ*m. (b) Quantification of MLI (*N* = 5, ANOVA, ^∗∗∗^*P* < 0.001). (c) Quantification of RAC (*N* = 5, ANOVA, ^∗∗∗^*P* < 0.001). (d) Comparison of pups' weight among four groups (*N* = 5, ANOVA). (e) Representative immunofluorescence images of vWF staining in the lung on PN14 in each group, scale bar = 100 *μ*m. (f) Quantification of vWF-positive vessels (<100 *μ*m) (*N* = 5, ANOVA, NS: not significant, ^∗∗∗^*P* < 0.001). (g) Lung resistance (LR) and (h) dynamic compliance (Cdyn) were determined from anesthetized and ventilated pups (*N* = 5, ANOVA, ^∗∗∗^*P* < 0.001). (i) RV/LV + *S* to measure RVH (*N* = 5, ANOVA, ^∗∗∗^*P* < 0.001).

After characterization of MVs, we next addressed the therapeutic effects of MVs in the IA-LPS BPD model, to determine whether MVs partly mediated the effects of hUCMSCs. Firstly, to determine the optimal MV concentration, we performed a series of dose–response experiments *in vivo*. MLI measurement on PN14 revealed that lung recovery was dependent on the concentration of the MVs administrated on PN7, and the peak was 20 *μ*g. Since there was no significant difference in MLI among 20 *μ*g, 40 *μ*g, or 80 *μ*g of MVs, we selected 20 *μ*g of MVs for the following *in vivo* experiments (Figures 4(a) and 4(b)). In the rat lung tissue, antenatal LPS-induced impaired alveolarization was significantly enhanced by transtracheal administration of MVs (Figure 5(a)). LPS exposure increased MLI and decreased RAC, but the effects were reverted after MV treatment (Figures 5(b) and 5(c)). Besides, there was a catch-up growth after transtracheal administration of MVs (Figure 5(d)). In terms of angiogenesis, however, MVs did not normalize the aberrant loss of small vessels caused by LPS (Figures 5(e) and 5(f)). MV treatment prevented loss of lung function, as shown by reduced lung resistance and increased lung compliance (Figures 5(g) and 5(h)). Increased RVH induced by LPS was also reversed by MVs (Figure 5(i)).

### 3.5. Duration and Localization of MVs *In Vivo*

**Figure 6:**
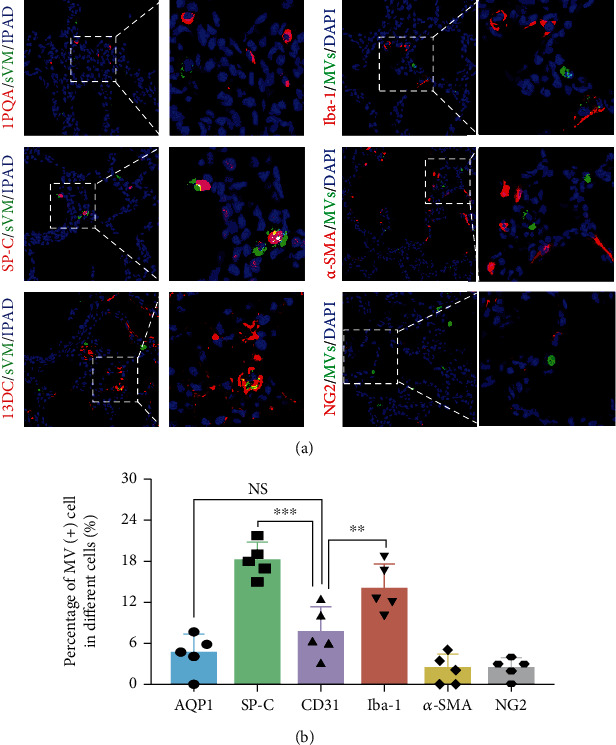
Location of the transtracheally delivered MVs according to the lung tissue cell types. (a) Representative immunofluorescence images of donor MVs and host lung cells staining at 48 hours after MV administration. The MVs were prestained with DiO dye (green). The nuclei were labeled with DAPI (blue); the AT1, AT2, vascular endothelial cells, total macrophages, vascular smooth muscle cells, and vascular pericytes were stained with AQP1, SP-C, CD31, Iba-1, *α*-SMA, and NG2 (red), respectively. (b) The rate of donor MV incorporation into each type of host lung cells (*N* = 5, ANOVA, NS: not significant, ^∗∗^*P* < 0.01 and ^∗∗∗^*P* < 0.001).

After addressing the therapeutic effects of MVs in the IA-LPS BPD model, we proceeded to examine the distribution of transplanted MVs in the lung tissue *in vivo*. Firstly, MVs were labeled with DiO green fluorescence and examined by immunofluorescence staining at different time points. The DiO green fluorescence gradually increased and reached a maximum at 48 hours postadministration, then gradually decreased, and finally disappeared after 96 hours (Fig. S1). Then, colocalization of DiO green fluorescence with various lung cell markers was examined by immunofluorescence staining at 48 hours (Figure 6(a)). DiO green fluorescence most frequently colocalized with SP-C-positive AT2 (18.2%) and was also identified with AQP-1-positive AT1 (4.5%), CD31-positive endothelial cells (7.6%), Iba-1-positive alveolar macrophages (14.0%), *α*-smooth muscle actin-positive smooth muscle cells (2.4%), and NG-2-positive pericytes (2.4%) (Figure 6(b)).

### 3.6. MVs Increase the Number of AT2 Cells and Attenuate Lung Inflammation in IA-LPS BPD Model Rats

**Figure 7:**
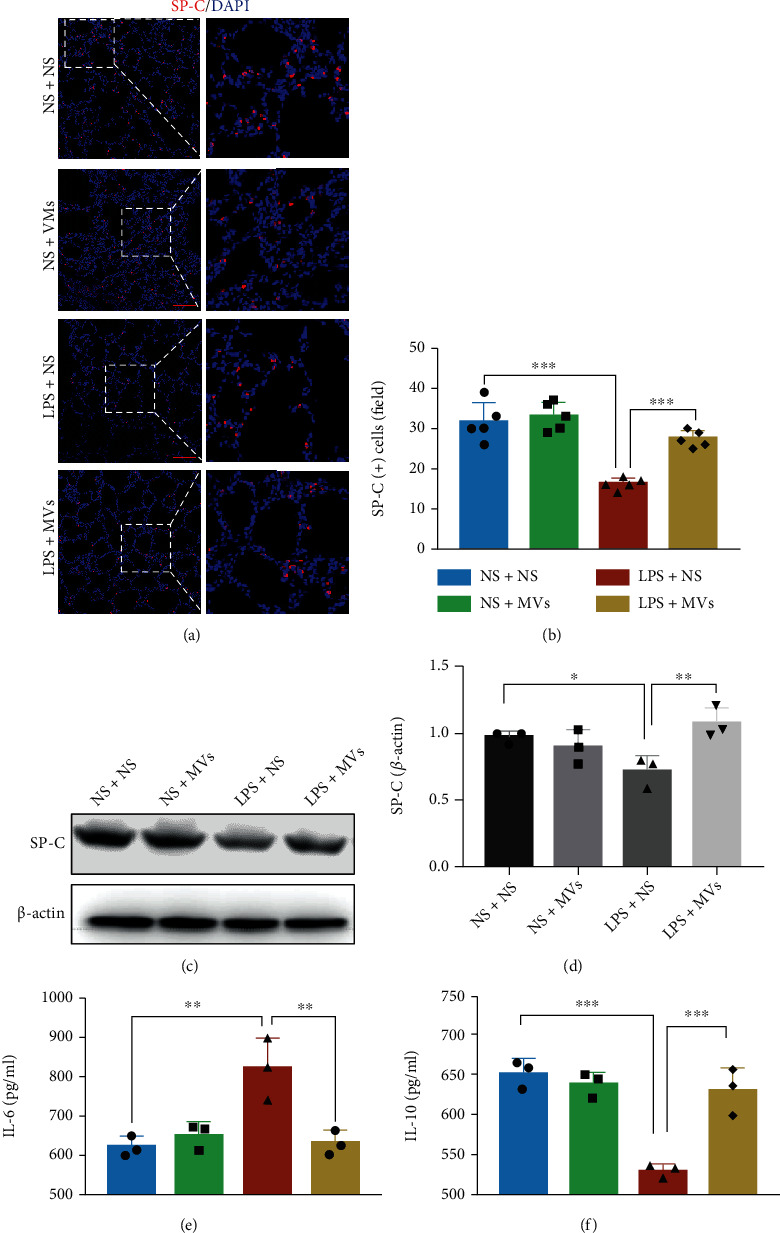
Effect of MVs on AT2 cells and macrophage-related inflammatory factors *in vivo*. (a) Representative immunofluorescence images of SP-C (red) staining in lung, scale bar = 100 *μ*m. (b) Quantification of SP-C-positive cells in each group (*N* = 5, ANOVA, ^∗∗∗^*P* < 0.001). (c) Western blot detection of protein levels of SP-C in each group. (d) Densitometric analysis was used to quantify the protein levels of SP-C (*N* = 3, ANOVA, ^∗∗∗^*P* < 0.001). The levels of (e) IL-6 and (f) IL-10 in lung homogenate were measured with ELISA (*N* = 3, ANOVA, ^∗∗^*P* < 0.01 and ^∗∗∗^*P* < 0.001).

Given that AT2 and lung macrophages were the two major cell types responsible for the uptake of MVs *in vivo*, we next investigated the effects of MVs on these two target cells. For AT2 cells, immunofluorescence staining of SP-C showed that LPS exposure decreased SP-C(+) cells, whereas MVs restored the number of SP-C(+) cells (Figures 7(a) and 7(b)). We also determined the protein level of SP-C, the major surfactant synthetized by AT2 cells. Antenatal LPS exposure decreased the expression of SP-C, which was partially reversed by MV treatment (Figures 7(c) and 7(d)). SP-A1, SP-B, and SP-D, the other three surfactants synthetized by AT2 cells, were also determined by WB; we found that they also decreased after LPS exposure, but they were not restored by MV treatment (Fig. S2).

Then, macrophage infiltration in the lung tissue was measured by Iba-1 immunofluorescence staining. Antenatal LPS exposure induced significant macrophage infiltration in the rat lung tissue (Fig. S3). In the treatment group, MVs significantly reduced macrophage infiltration in comparison with the LPS group (Fig. S3). IL-6 (proinflammatory cytokine) and IL-10 (anti-inflammatory cytokine), major inflammation mediators released by macrophages, were measured by ELISA. The concentration of IL-6 was increased, while IL-10 was decreased in lung homogenates of LPS-exposed rats, and these responses were significantly reversed by transtracheal administration of MVs (Figures 7(e) and 7(f)). Taken together, MVs from hUCMSCs have anti-inflammatory effects on the IA-LPS BPD model *in vivo*.

### 3.7. MVs Improve MLE-12 Cell Proliferation following LPS-Induced Injury *In Vitro*

**Figure 8:**
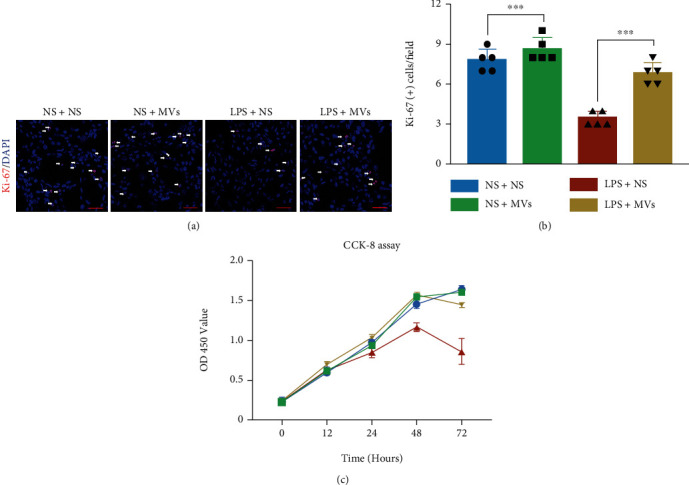
MVs improve MLE-12 cell proliferation following LPS-induced injury. (a) Representative immunofluorescence images stained by Ki-67 (red) in MLE-12 cells, scale bar = 100 *μ*m. (b) Quantitative analysis of Ki-67-positive cells in MLE-12 cells treated by LPS with or without MVs for 48 hours (*N* = 5, ANOVA, ^∗∗∗^*P* < 0.001). (c) CCK-8 analysis of MLE-12 cells' viability (*N* = 5, ANOVA).

Given that AT2 cells are the stem cells in the newborn lung and play a role in the alveolar structure involved in pulmonary respiratory function [36], we further investigated the effects of MVs on MLE-12 cells *in vitro*. MLE-12 cells were treated by LPS with or without MVs for 48 hours. Then, they were evaluated by Ki-67 and Annexin V/PI staining. Ki-67 staining showed that LPS exposure significantly reduced the proliferation rate of MLE-12 cells, which was improved to normal level by MV treatment (Figures 8(a) and 8(b)). The flow cytometry analysis showed that LPS did not alter the apoptotic rate of MLE-12 cells (data not shown). The CCK-8 assay results further illustrated that MV treatment improved the LPS-induced decrease in MLE-12 cells' survival (Figure 8(c)).

### 3.8. MVs Improve Alveolarization and Attenuate Lung Inflammation Associated with the PTEN/AKT and the MAPK Pathways

**Figure 9:**
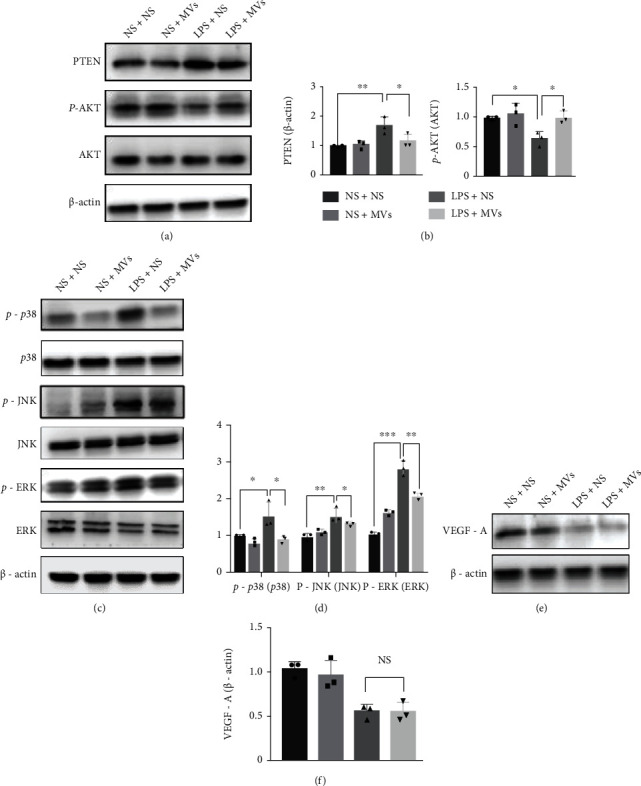
MVs activate the PTEN/p-AKT pathway and suppress the MAPK pathway in the IA-LPS BPD model. (a) The protein levels of PTEN, p-AKT at S473, and AKT were evaluated by western blotting on lung homogenates on PN14. (b) Densitometric analysis was used to quantify the expression of PTEN and p-AKT/AKT (*N* = 3, ANOVA, ^∗∗^*P* < 0.01 and ^∗∗∗^*P* < 0.001). (c) The protein levels of p38, JNK, ERK, and their phosphorylated forms (p-p38, p-JNK, and p-ERK) were evaluated by western blotting on lung homogenates on PN14. (d) Densitometric analysis was used to quantify the phosphorylation of p38 at Thr180/Tyr182, JNK at Thr183/Tyr185, and ERK1/2 at Thr202/Tyr1204 (*N* = 3, ANOVA, ^∗∗^*P* < 0.01 and ^∗∗∗^*P* < 0.001). (e) The protein levels of VEGF-A in lung homogenate were evaluated by western blotting on PN14. (f) Densitometric analysis was used to quantify the expression of VEGF-A (*N* = 3, ANOVA, NS: not significant, ^∗∗∗^*P* < 0.001).

Subsequently, we explored the specific mechanism by which MVs improve alveolarization and attenuate lung inflammation in the IA-LPS BPD rat model. The level of PTEN significantly increased, and p-AKT expression remarkably decreased in rat lungs from the IA-LPS BPD rats compared with the control group. MV treatment reversed the protein levels of both PTEN and p-AKT (Figures 9(a) and 9(b)). Meanwhile, the results of WB showed that LPS exposure increased the expression levels of p-p38, p-JNK, and p-ERK, while MV treatment partially suppressed p-p38, p-JNK, and p-ERK expression after antenatal LPS exposure (Figures 9(c) and 9(d)). However, the expression of VEGF-A suppressed by antenatal LPS exposure was not restored by MV administration (Figures 9(e) and 9(f)).

## 4. Discussion

Although there is not yet effective treatment for BPD, the advent of MSCs provides new hope for BPD treatment. Many preclinical studies have demonstrated that MSCs and small extracellular vesicles (or exosomes) have a protective effect on lung injury on BPD [21, 28, 37, 38]. Furthermore, most clinical trials of MSCs in BPD are at a phase I stage, demonstrating the safety of stem cell therapy in human. However, the therapeutic effects and function of large extracellular vesicles (or MVs) derived from MSCs on BPD are poorly understood.

In this study, we showed that the MVs derived from hUCMSCs enhanced alveolar development and alleviated lung inflammation in the IA-LPS BPD model, and this protection was associated with the PTEN/AKT pathway and the MAPK pathway. These findings also suggest that MVs are key paracrine therapeutic mediators of hUCMSCs and show potential for application in safe and effective cell-free therapy for BPD associated with antenatal factors.

Many studies have shown that antenatal factors are strongly associated with susceptibility to BPD. However, the most commonly used animal model for BPD research involves exposure to postnatal hyperoxia; thus, it cannot reflect antenatal factors that influence BPD. Here, we used a rat model of BPD induced by intra-amniotic injections of LPS; LPS induced impaired alveolarization and diminished lung function, particularly mimicking BPD of preterm infants in humans [39, 40].

Functions of EVs depend on their ability to interact with recipient cells to deliver their contents (proteins, lipids, and RNAs) [41]. Our data showed that MVs were mainly uptaken by AT2 cells (18.2%) and macrophages (14.0%) and were rarely observed in vascular endothelial cells (7.6%) and vascular pericytes (2.4%). However, our findings are not fully consistent with the previous report that exosomes were mostly uptaken by vascular pericytes (22.7%), AT2 cells (19.5%), and macrophages (21.3%) [37]. The difference in the cellular uptake ability of exosomes and MVs in vascular pericytes might be related to the difference in the physicochemical properties of these two types of EVs, such as origin, size, morphology, and buoyant density [42].

AT2 cells have critical secretory and regenerative roles in the alveoli to maintain lung homeostasis [36]. Our data showed that MVs diminished most of the effects of antenatal LPS-injury on AT2, suggesting similar therapeutic effects to those of exosomes on AT2 in hyperoxia-induced BPD model [21]. Additionally, there are many other lung stem cells, such as bronchioloalveolar stem cells, Clara cells, basal cells, and distal airway stem cells [43, 44]. One study has reported that MSCs increase bronchioloalveolar stem cells in hyperoxia-induced bronchopulmonary dysplasia [45]. Further studies should investigate the effects of MVs on different lung stem cells.

As another major target cell of MVs, lung macrophages are dominant immune cells in the inflammation [46]. Our results showed that antenatal LPS induced macrophage infiltration, and this response was restored by MV treatment, which decreased the proinflammatory factor IL-6 and increased the anti-inflammatory factor IL-10 to normal level. Several studies have noted that pulmonary macrophages occupy an “M2-like” phenotype, which can persist for several months in BPD [29, 47]. Due to the anti-inflammatory action of MVs, further studies should examine whether MVs could modulate the dysregulated macrophage phenotype in an experimental BPD model.

It has been shown that the proliferation of AT2 cells in lung injury is linked to abnormal expression of PTEN/AKT [48]. As a major proliferation-linked signaling pathway, the PTEN/AKT pathway was examined in our study. Our data showed that MV treatment reversed the protein levels of PTEN and p-AKT, suggesting that the AT2 cell proliferation promoted by MVs may be related to the PTEN/AKT pathway.

Furthermore, previous studies have shown that the antenatal exposure of preterm infants to infection and inflammation may result in adverse fetal consequences, such as BPD [49]. Activation of MAPK signaling is important in the response to inflammation [50]. The signaling mediators of MAPK include ERKs, JNKs, and p38 MAPK [51]; however, their role in antenatal LPS-induced lung injuries has not been identified. Our results showed that the expression levels of p-p38, p-JNK, and p-ERK were significantly increased but were suppressed by MV administration. These data suggested that MVs alleviate LPS-induced lung injuries by a mechanism associated with the suppressed MAPK pathway. In addition, MVs were ineffective at promoting the expression of VEGF-A, which might be the reason why MVs did not restore pulmonary microvasculature; however, this aspect deserves further investigation.

Since exosomes received most of the attention, the effect of MVs is poorly understood before. Our results show that MVs have the potential to exert both proinflammatory and proproliferation actions, providing a detailed framework for the successful use of MVs as a strategy for BPD treatment. These interesting results deserve more research efforts in the future. Our study also has the potentiality to be applied on other repairing strategies with novel biomedical materials, like the Phosphorene or the Borophene [52].

Although encouraged by these findings, we acknowledge several limitations of this study. First, the concentration and duration of LPS in amniotic sac, as well as intake of LPS in the lung, were not examined. Second, we only administered MVs in IA-LPS BPD model rats. Other studies have shown that exosomes have similar therapeutic effects as MSCs [53]. Future studies should consider comparing the effects of different EV subsets on experimental BPD models in a more direct way. Indeed, a single active ingredient has not been further investigated in this study. Rather, MVs likely provide an orchestra of bioactive components that function synergistically to play a therapeutic role. Furthermore, because IA-LPS BPD model focused on antenatal factors, the findings of this study need to be interpreted in the context of the experimental model.

## 5. Conclusion

In conclusion, we demonstrated that MVs derived from hUCMSCs restored lung architecture and function and improved RVH in the IA-LPS BPD model by promoting AT2 cell proliferation and attenuating lung inflammation. The underlying mechanism was associated with the PTEN/AKT pathway and the MAPK pathway. Our findings may offer a new perspective for the treatment of BPD by MVs.

## Data Availability

The authors confirm that all data underlying the findings are fully available upon request.

## Supplementary Materials

Supplementary Materials Figure S1: *in vivo* imaging of MVs in the IA-LPS BPD model. (A) Representative fluorescence images of IA-LPS BPD rats after treatment with MVs at selected time points, scale bar = 10 *μ*m. (B) The number of DiO-positive cells in the lungs subjected to transtracheal injection of MVs at selected time intervals (*N* = 5, ANOVA, NS: not significant, ^∗∗∗^*P* < 0.001). Figure S2: effect of MVs on pulmonary surfactants. (A) Western blot detection of protein levels of SP-A1, SP-B, and SP-D in each group. (B) Densitometric analysis was used to quantify the protein levels of SP-A1, SP-B, and SP-D in each group (*N* = 3, ANOVA, NS: not significant, ^∗∗∗^*P* < 0.001). Figure S3: effect of MVs on lung macrophage infiltration. (A) Representative immunofluorescence images of Iba-1 (red) staining in lung tissue. (B) Quantification of Iba-1-positive cells in each group (*N* = 5, ANOVA, ^∗∗∗^*P* < 0.001).
