# Supplementary figures and images for: Microvesicles Derived from Human Umbilical Cord Mesenchymal Stem Cells Enhance Alveolar Type II Cell Proliferation and Attenuate Lung Inflammation in a Rat Model of Bronchopulmonary Dysplasia
Source: Stem Cells Int. 2022 Jun 27;2022:8465294. doi: 10.1155/2022/8465294 (PMC9252687; doi:10.1155/2022/8465294)

# Figure S1

**A**

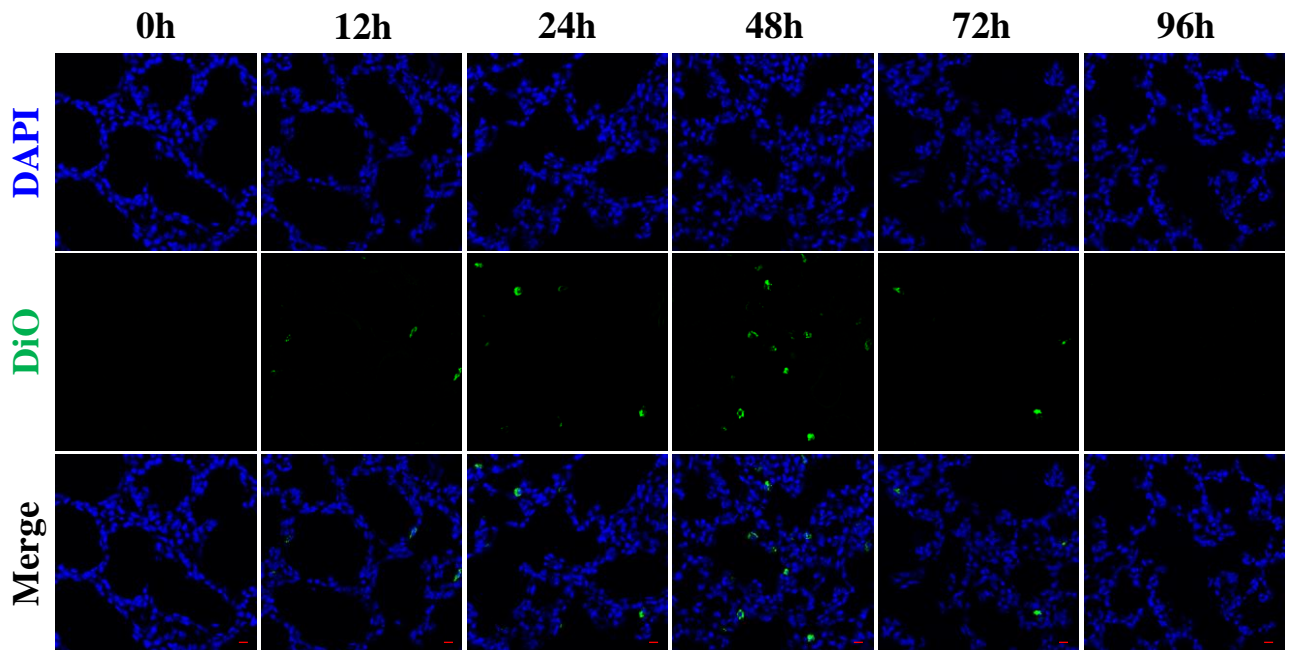

**B**

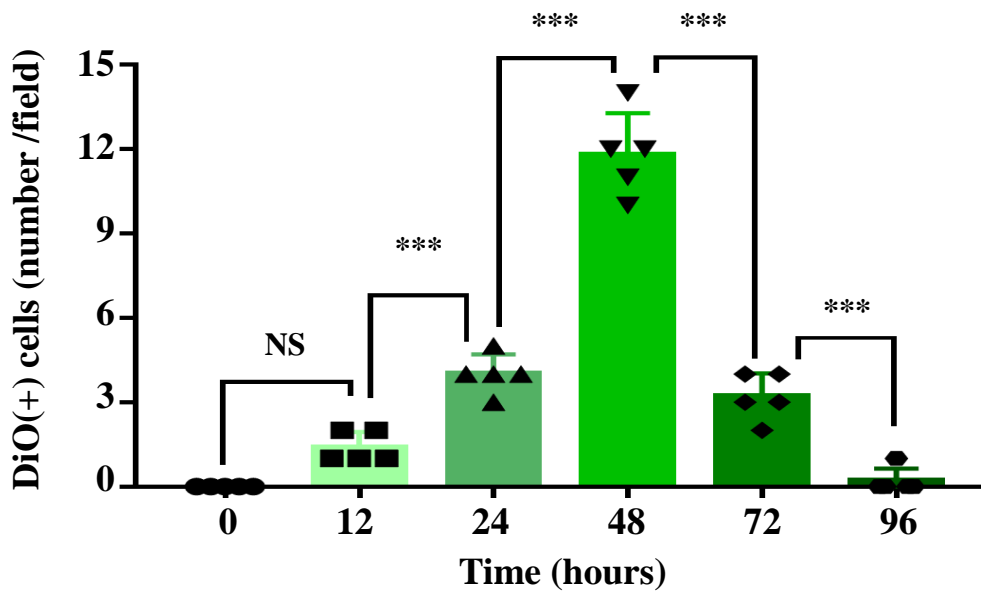

# Figure S2

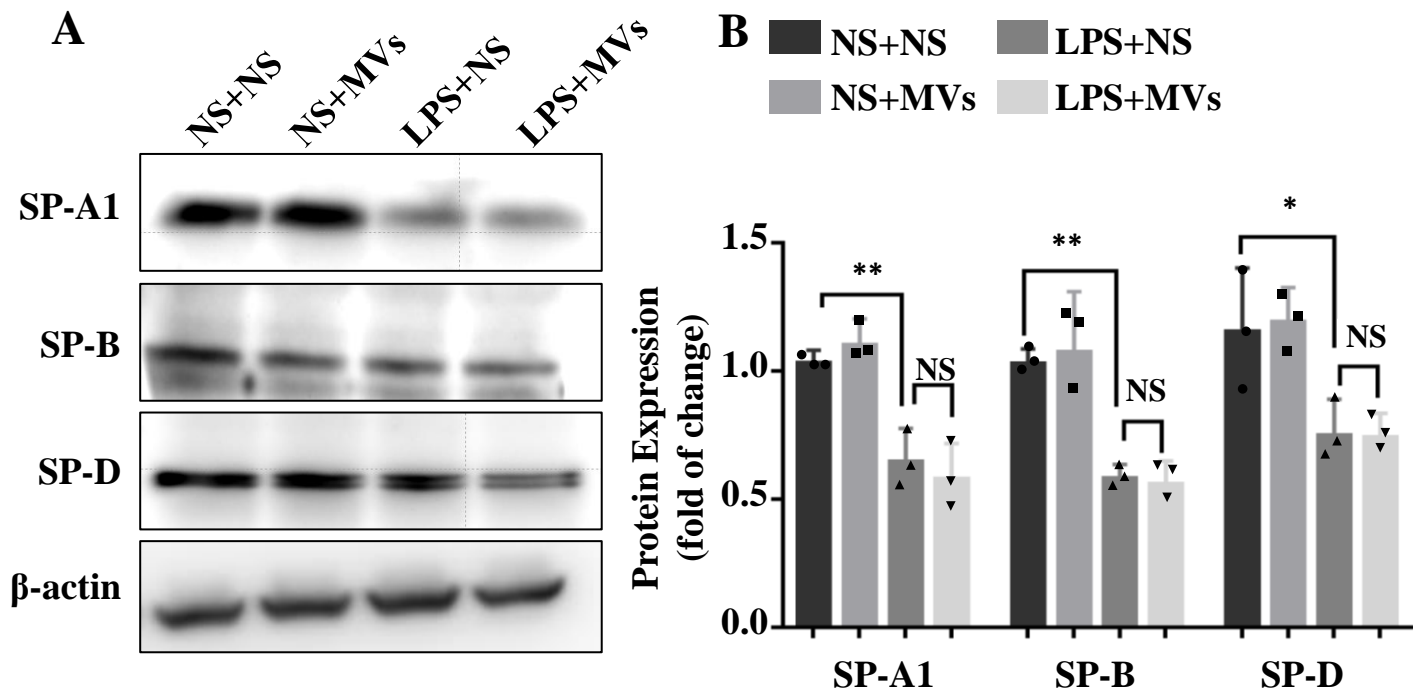

# Figure S3

**A**

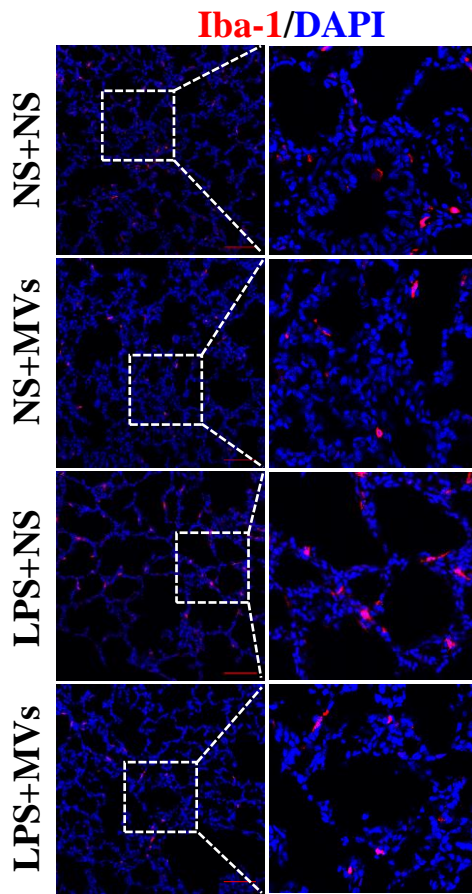

**B**

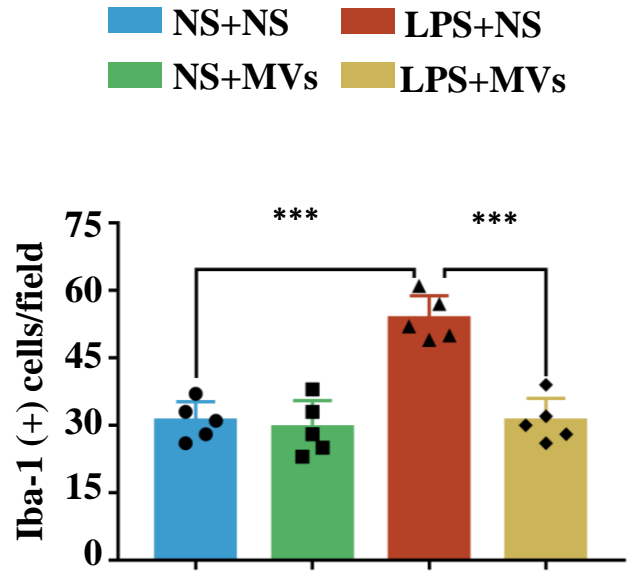

Supplement: Supplementary Materials — Figure S1: in vivo imaging of MVs in the IA-LPS BPD model. (A) Representative fluorescence images of IA-LPS BPD rats after treatment with MVs at selected time points, scale bar = 10 μm. (B) The number of DiO-positive cells in the lungs subjected to transtracheal injection of MVs at selected time intervals (N = 5, ANOVA, NS: not significant, ∗∗∗P < 0.001). Figure S2: effect of MVs on pulmonary surfactants. (A) Western blot detection of protein levels of SP-A1, SP-B, and SP-D in each group. (B) Densitometric analysis was used to quantify the protein levels of SP-A1, SP-B, and SP-D in each group (N = 3, ANOVA, NS: not significant, ∗∗∗P < 0.001). Figure S3: effect of MVs on lung macrophage infiltration. (A) Representative immunofluorescence images of Iba-1 (red) staining in lung tissue. (B) Quantification of Iba-1-positive cells in each group (N = 5, ANOVA, ∗∗∗P < 0.001). [file 8465294.f1.pdf]
